# Dimensions of leisure-time physical activity and risk of depression in the “Seguimiento Universidad de Navarra” (SUN) prospective cohort

**DOI:** 10.1186/s12888-020-02502-6

**Published:** 2020-03-04

**Authors:** Alejandro Fernandez-Montero, Laura Moreno-Galarraga, Almudena Sánchez-Villegas, Francisca Lahortiga-Ramos, Miguel Ruiz-Canela, Miguel Ángel Martínez-González, Patricio Molero

**Affiliations:** 1 Department of Occupational Medicine, University of Navarra Clinic, Av. Pio XII, 36, 31008 Pamplona, Navarra Spain; 2 Department of Preventive Medicine and Public Health, University of Navarra, Pamplona, Spain; 3 IdiSNA (Instituto de Investigación Sanitaria de Navarra), Pamplona, Spain; 4 Department of Pediatrics, Complejo Hospitalario de Navarra, Servicio Navarro de Salud, Pamplona, Spain; 5 Nutrition Research Group, Research Institute of Biomedical and Health Sciences, University of Las Palmas de Gran Canaria, Las Palmas de Gran Canaria, Spain; 6 CIBER Fisiopatología de la Obesidad y Nutrición (CIBER Obn), Instituto de Salud Carlos III, Madrid, Spain; 7 Department of Psychiatry and Medical Psychology, University Clinic of Navarra, Pamplona, Spain; 8 Department of Nutrition, Harvard TH Chan School of Public Health, Boston, USA

**Keywords:** Physical activity, Lifestyle, Depression, Health promotion, SUN

## Abstract

**Background:**

An inverse association between total leisure-time physical activity (LTPA) and depression has been previously documented in the scientific literature. Our objective was to prospectively assess the association of LTPA with the risk of depression, focusing on several dimensions of LTPA (intensity, duration and type).

**Methods:**

The SUN (Seguimiento Universidad de Navarra) project is a prospective cohort study formed by Spanish university graduates. A total of 15,488 adults (40.2% men, mean age 37 ± 12 years) initially free of depression were assessed. A report of a validated medical diagnosis of depression or the habitual use of antidepressants (any of both) were considered as incident cases of depression. LTPA was estimated through previously validated self-reported questionnaires. Participants were classified following Physical Activity recommendations from the World Health Organization, and according to the intensity, duration and type of LTPA. Cox proportional hazards regression models were run, adjusted for demographic, lifestyle, and dietary factors, to estimate adjusted hazard ratios (HR) of depression and 95% confidence intervals (CI).

**Results:**

During 163,059 person-years of follow-up we registered 870 incident cases of depression. Participants with higher total LTPA (METs-h/wk) and higher duration of LTPA (hours/wk) exhibited a lower risk of depression HR = 0.84 (95% CI: 0.72–0.99) and HR = 0.83 (0.70–0.99) respectively, whereas intensity of LTPA (MET) did not show any association with depression.

**Conclusion:**

Participants with higher LTPA had a lower risk of depression. The inverse association was stronger for total LPTA time than for its intensity. Higher duration of LTPA should be encouraged to prevent depression.

## Background

Depression, a mental disorder affecting over 300 million people [1] is the third leading cause of global burden of disease. By 2030 depression will represent the largest contributor to the burden of disease in high–income countries [2].

Multiple factors have been implicated in depression genesis, among them, important potentially modifiable lifestyle risk factors such as poor dietary habits, low education, cigarette smoking, high body mass index, sedentary lifestyles and reduced level of physical activity [3, 4].

Depression shares similar etiologic mechanisms and risk factors with metabolic syndrome, diabetes mellitus and cardiovascular disease (CVD) [5–7]. Therefore, CVD and depression, considered the most frequent diseases worldwide [8] seem to be vulnerable to similar preventive approaches. Secular humankind experience convincingly shows that the most effective mean to reduce the population burden of disease is by learning how to prevent diseases that are responsible for the greatest part of that burden [9]. The selection of lifestyle factors that can protect against the most prevalent diseases seems to be a highly efficient approach; this is apparently the case of leisure-time physical activity (LTPA).

Different studies have been published reinforcing the favorable and consistent effects of LTPA on cardiometabolic disease [10, 11] and cancer [12]. LTPA has beneficial effects over mental health diseases, such as depression, anxiety and stress, and also over quality of life [13, 14]. LTPA increases cortical blood flow, endorphins and epinephrine production, and influences the dopamine, noradrenaline and serotonin systems. In addition, it may act against pro-inflammatory pathways behind the common mechanisms involved in CVD and depression [15].

The World Health Organization (WHO) has adopted the *Comprehensive mental health action plan 2013–2020* and one of its four major objectives is to implement strategies for the promotion of mental health [16]. Based on previous evidence, the WHO published specific recommendations on physical activity (PA). According to these recommendations, adults aged 18–64 should engage in at least 150 min of moderate-intensity or at least 75 min of vigorous-intensity aerobic PA throughout the week, to promote a physically and mentally healthy lifestyle [17].

The beneficial effect of PA on depression has been previously studied. Small intervention studies assessing the amelioration of pre-existent depression suggested that physical activity could be beneficial when treating adults with depression, [18] and the Cochrane Collaboration in a 2013 review, concluded that physical activity had clinical effects on depressive patients [19].

The WHO recommendation represents an unspecific combination of time and intensity. A number of prospective epidemiologic studies have already shown an inverse association between total LTPA and the risk of incident depression [20]. However, the independent effect of the duration, type and intensity level of LTPA deserves further investigation.

The aim of this study was to investigate the association of LTPA intensity (measured in Metabolic Equivalent for Task (MET)), the LTPA duration (measured in hours/week), the total LTPA (measured in METS-hour/week), and different types of LTPA with depression incidence.

## Methods

### Study population

The SUN Project (Seguimiento Universidad de Navarra) is a Mediterranean prospective multipurpose dynamic cohort formed by Spanish graduates. The objectives, design and methods have been previously described [21]. Briefly the recruitment started in December 1999 and it is permanently open. The objectives are to identify dietary and lifestyle determinants of metabolic diseases, CVD, mental diseases, cancer and other chronic conditions. Data collection and follow up is carried out through mailed or web-based biennial self-reported questionnaires. More information and cohort profile can be found in the web page: www.medpreventiva.es/xZd6Hh

The baseline questionnaire (QO) collects information on participants’ socio-demographics, anthropometrics, diet and eating behaviors, lifestyles and clinical data. Every 2 years, shorter follow-up questionnaires (Q2-Q16) track changes in diet, lifestyle and medical aspects and ascertain the incidence of new diseases.

**Fig. 1:**
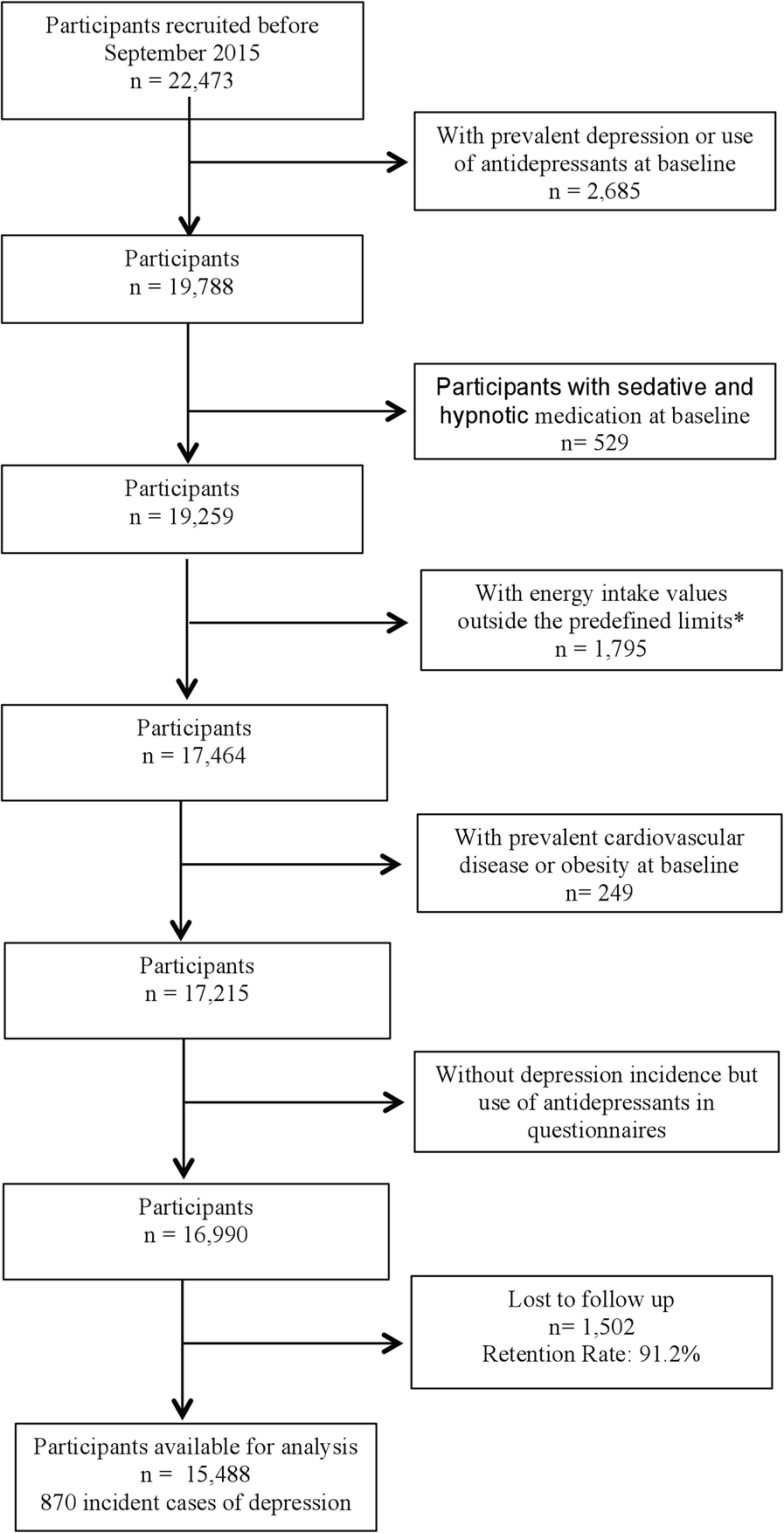
Flow-chart. The SUN Project 1999–2018 * Willet 2012

Twenty-two thousand four hundred seventy-three participants were recruited before September 2015. Appropriate exclusions for prevalent depression and other factors are shown in the flow chart in Fig. 1. We excluded participants with sedative and hypnotic medication, participants with energy intake values outside the Willet limits and participants with prevalent cardiovascular disease or obesity at baseline, which might affect the capacity to perform PA. After exclusions, 15,488 participants were included with a mean follow-up period of 10.5 years (SD: 4.4).

The written completion of the baseline questionnaire or Q0, once participants understood the specific information needed, the methods used to deliver their data and the future feedback from the research team, was considered to imply informed consent. Information explaining this was included in the Q0. We specifically asked for written permission before follow-up of personal medical records. We informed the potential candidates of their right to refuse to participate in the SUN study or to withdraw their consent to participate at any time without reprisal, according to the principles of the Declaration of Helsinki. The Institutional Review Board of the University of Navarra approved this survey and methods.

### Assessment of leisure time physical activity

LTPA has been defined by WHO as the physical activity realized by an individual not required as an essential activity of the daily routine, excluding therefore sports participation, exercise conditioning, or other recreational activities [17].

The time spent and type of LTPA used in this analysis was derived from a 17-item questionnaire included in the Q0, collecting information about 17 different sports or leisure time activities (walking, jogging, athletics, cycling, stationary cycling, swimming, tennis, soccer, basketball, dancing, hiking, gymnastics, gardening, skiing, martial arts and sailing,) and information about the time spent on each LTPA. This questionnaire was previously validated using triaxial accelerometers (Spearman’s ρ 0.51 (95%CI: 0.23–0.70)) [22].

To calculate the energy expenditure in LTPA, we multiplied the weekly hours dedicated to each activity (Time spent in LTPA) by its typical intensity expressed in metabolic equivalents (METs) [21] to obtain METs-h / wk. Then, we divided the total sum of METs-h / wk. by the weekly hours of LTPA to generate an average of METs (intensity in LTPA).

We classified LTPA energy expenditure in three groups, according to the WHO “Global recommendation on Physical Activity for Health” for adults aged 18–64 [17]: a) below the WHO recommendation (< 10 MET-h/wk) equivalent < 150 min/wk. of moderate-intensity aerobic PA or < 75 min/wk. of vigorous-intensity aerobic PA throughout the week or an equivalent combination; b) amount suggested by the WHO (10 to 20 METs-h/wk.; equivalent to between 150 and 300 min/wk. of moderate-intensity aerobic PA or 75–150 min/wk. of vigorous-intensity aerobic PA or an equivalent combination; c) above the WHO recommendation (> 20 MET-h/wk) equivalent to > 300 min/wk. of moderate-intensity or more than 150 min/wk. of vigorous-intensity PA per week or an equivalent combination.

### Outcome assessment: depression

All follow-up questionnaires included the question: *Has a physician diagnosed you of depression in the last 2 years?* Participants, who self-reported the diagnosis of depression or the use of antidepressants were considered as incident cases of depression. This procedure for the ascertainment and adjudication of medically diagnosed depression cases in the SUN Project has been previously validated using structured clinical interviews. The validation study in a subsample of the cohort used the Structured Clinical Interview for DSM-IV (SCID-I) administered by a senior psychiatrist as Gold Standard blinded to the responses of the questionnaire. The percentages of depression cases and confirmed non-depression cases both by a psychiatrist were 74.2% (95% CI 63·3–85·1) and 81.1% (95% 69·1,92·9) respectively [23].

### Assessment of covariates

At baseline (Q0), information of potential confounding factors such as personality traits, health-related habits (sleeping, smoking, alcohol intake, use of nap and time watching TV), prevalent chronic disease (hypertension, diabetes mellitus and cancer), dietary factors (adherence to Mediterranean diet, special diets and total energy intake), validated self-reported anthropometric data (weight and height) and socio-demographic characteristics (sex, age and educational level), were collected. To assess personality traits, validated specific self-reported questions, rated 0–10 regarding competitiveness, level of tension, and dependency, were used, using psychometric Likert-type scales [23]. For measuring dietary factors, we used a 136-item semi-quantitative food frequency questionnaire, previously validated [24]. We classified participants according to their baseline adherence to Mediterranean diet according to the validated 14-item PREDIMED questionnaire [25].

### Statistical analysis

To analyze the contribution of each type of PA to the total energy expenditure in LTPA, we calculated the percentage of total variability (R^2^) in the amount of MET-h/wk. accounted for, by each type of PA.

Person-years of follow-up was calculated from the date baseline questionnaire was completed to the date of depression diagnosis, death, or return of the last follow-up questionnaire, whichever occurred first. To analyze depression risk according to LTPA categories proposed by the WHO we estimated hazard ratios (HR) and their 95% confidence intervals (CI) using Cox regression models with age as the underlying time variable. We used the lowest LTPA category (< 10 MET-h/wk) as the reference category and adjusted for multiple confounding factors (sex, baseline body mass index, time sleeping, time of siesta, time spent watching television, total energy intake, adherence to the Mediterranean Diet, alcohol intake, smoking, educational level, and history of hypertension, diabetes mellitus and cancer). To asses possible changes during follow up in participants PA levels, the specific question: *Has your level of PA increased, remained the same or decreased in the last 2 years?* was included in the follow-up questionnaires. This information was taken into account in the multivariable analysis. We used categories of age (decades of age) and calendar year of entering the cohort as stratification variables. Linear trend was analyzed by assigning to each category of energy expenditure the median of that category in METs-h/wk. and including this continuous variable in the multivariable models. We repeated these analyses, adjusting for multiple comparisons, for each of the 17 different types of LTPA.

The same analyses were conducted for the time spent in LTPA per week and for LTPA -intensity, using < 75 min/wk. and inactive, respectively, as reference categories.

Nelson-Aalen curves were used to describe the incidence of depression in different categories of LTPA and applied to these curves inverse probability weighting methods to control for confounding. To build these weights we used multinomial logistic regression models with LTPA (3 categories) as the outcome and derived the conditional probabilities for each subject to be in each category according to potential confounders. Subsequently we calculated stabilized weights as the quotient between the marginal probability of being in that category of LTPA over the conditional probability derived from the multinomial logistic model.To ensure the chronological sequence, and confirm that LTPA temporally preceded depression incidence, supplementary analysis have been done eliminating participants with a diagnosis of depression not only at baseline(Q0) but also in the first follow up questionnaire (Q2). Stratified analyses and tests for interactions with sex, age and adherence to the Mediterranean diet were performed to ensure the robustness of the results in different scenarios. All *p* values presented are two-tailed; *p* < 0.05 was considered statistically significant. Analyses were performed using STATA 12.0.

## Results

During follow-up (mean 10.5 years, SD: 4.44) 870 incident cases of depression were identified over a total of 163,059 person-years.

Supplemental table shows the contribution of each one of the 17 different types of physical activity to the total energy expenditure in LTPA. The principal moderate activities (walking and stair climbing) contributed more than 50% to the quantity MET-h/wk. Activities more vigorous, such as fitness, jogging or athletics, had a greater impact in total LTPA variability.

The distribution of baseline characteristics according Physical Activity are summarized in Table 1. Subjects with higher LTPA were more likely to be men, smoke less and had higher alcohol consumption. They also were more competitive and practiced physical activity during a longer time and more vigorously.

**Table 1:** Baseline characteristics of participants according to the WHO recommended levels of physical activity for adults aged 18–64 years (MET-h/week). The SUN Project 1999–2018

		< 10 MET-h/wk	10 to 20 MET-h/wk	> 20 MET-h/wk
**N**		5597	3548	6343
**Age (years)**		37.5 (11.7)	36.6 (11.9)	36.6 (12.1)
**Women (%)**		66.9	61.3	52.6
**Education Level (%)**	**College**	24.6	24.6	22.9
	**Post grade**	49.8	48	48.3
	**Master**	7.2	8.2	8.7
	**Doctorate**	9.5	10.4	10.0
**Sleep (h/d)**		7.4 (1.0)	7.4 (1.0)	7.4 (1.0)
**Siesta (mid-day nap) (h/d)**		0.5 (.5)	0.5 (0.5)	0.6 (0.5)
**Average time watching TV (h/d)**		1.7 (1.4)	1.6 (1.3)	1.5 (1.2)
**Body mass index (kg/m**^**2**^**)**		23.6 (3.7)	23.4 (3.4)	23.4 (3.2)
**Total energy intake (kcal/d)**		2309 (611)	2321 (601)	2405 (621)
**MEDAS (14-itens)**		5.4 (1.8)	5.5 (1.8)	5.8 (1.9)
**Use of special diets (%)**		6.36	6.91	8.48
**Alcohol intake (g/d)**		6.0 (10.2)	6.3 (9.3)	7.2 (9.6)
**Smoking pack-years**		6.1 (10.7)	4.9 (9.1)	4.9 (9.1)
**Hypertension**^**a**^**(%)**		9.17	8.85	8.75
**Diabetes mellitus**^**b**^**(%)**		1.72	1.32	1.72
**Cancer (%)**		3.39	3.07	3.69
**Competitiveness level**^**c**^		6.8 (1.8)	7.0 (1.7)	7.1 (1.6)
**Psychological tension level**^**c**^		5.9 (2.2)	5.8 (2.1)	5.8 (2.1)
**Dependency level**^**c**^		3.6 (2.8)	3.7 (2.8)	3.5 (2.9)
**Leisure-time physical activity hours (hours/week)**		1.8 (1.8)	3.1 (0.8)	8.0 (4)
**Average METs**		4.6 (1.1)	5.0 (1.1)	5.2 (1.1)
**Changes in physical activity**^**d**^	**No change**	47.2	42.1	41.1
	**Increased**	35.7	36.9	37.7
	**Decreased**	17.1	21.0	19.7

^a^ Or antihypertensive treatment

^b^ Or antidiabetic treatment

^c^ Scale (0–10)

^d^ Reported in the 2th and 4th year follow-up questionnaires

*MET* metabolic equivalents

*MEDAS* Mediterranean Diet Adherence Screener questionnaire

**Fig. 2:**
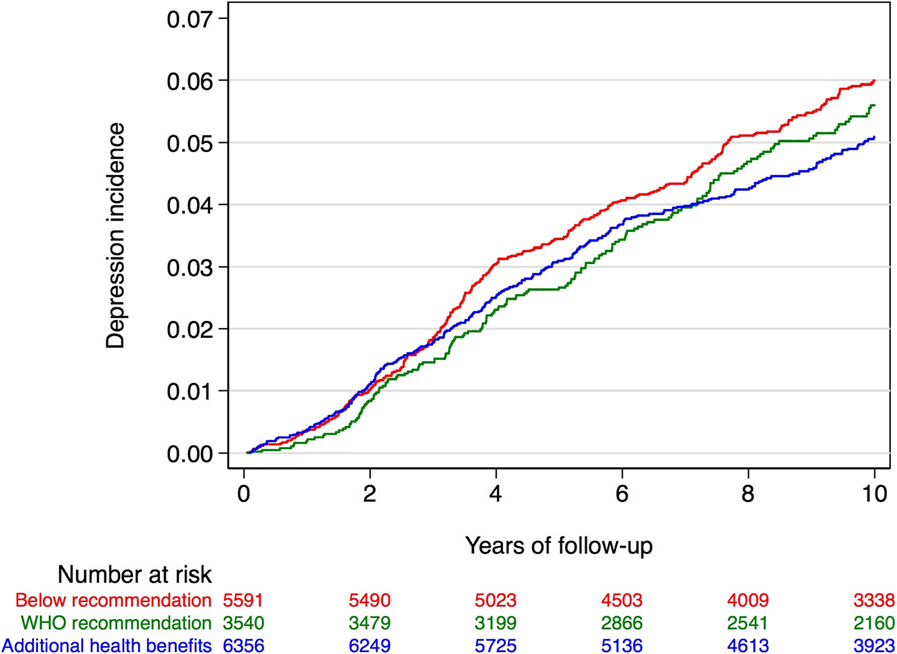
Nelson-Aalen curves for the incidence of depression over time, across WHO categories of total LTPA according to actual WHO recommendations for adults. The SUN prospective cohort. Adjusted for confounding using inverse probability weighting

Figure 2 shows weighted Nelson-Aalen curves for the incidence of depression over time, across the three categories of total LTPA, following the updated classification by WHO recommendation (group 1: Below recommendation, group2: in the recommendation range, and group 3: over WHO recommendation) show that the biggest different appear between subjects that follow the WHO recommendation on Physical Activity compared with subjects under this recommendation.

Table 2 shows the risk of depression according to total LTPA quantity (intensity and duration in Met-h per week), LTPA time (hours/week) and LTPA intensity (METs). Higher total LTPA and higher duration were inversely associated with the risk of depression, whereas intensity of LTPA did not show any relationship with depression incidence.

**Table 2:** Depression risk according to different dimensions of baseline physical activity. The SUN Project 1999–2018

Total leisure-time physical activity (MET-h/wk)				< 10 MET-h/wk
Depression cases / person-years	366/59193	10 to 20 MET-h/wk	> 20 MET-h/wk	P for trend
Multivariable-adjusted HR ^a^ (95% CI)	1 (ref.)	193/37606	311/66260	
**Time spent in leisure time physical activity (hours/wk)**	**< 75 min/wk**	0.88 (0.74–1.05)	0.84 (0.72–0.99)	0.046
Events / person-years	279/44420	**75–300 min/wk**	**> 300 min/wk**	
Multivariable-adjusted HR ^b^ (95% CI)	1 (ref.)	345/65624	246/53015	
**Intensity in leisure time physical activity (average METS)**	**Inactive**	0.88 (0.75–1.04)	0.83 (0.70–0.99)	0.057
Events / person-years	161/26004	**< 6 Average METs**	**≥6 Average METs**	
Multivariable-adjusted HR ^c^ (95% CI)	1 (ref.)	574/110834	135/26220	

^a^ Adjusted for sex, baseline body mass index, time sleeping, time nap, time TV, total energy intake, adherence to the Mediterranean Diet, alcohol intake, smoking pack years, educational level, hypertension, diabetes mellitus, cancer and changes in physical activity in the 2th and 4th year follow-up, with age and year of entering the cohort as stratification variables

^b^ Additional adjusted by intensity physical activity

^c^ Additional adjusted by leisure time physical activity

**Fig. 3:**
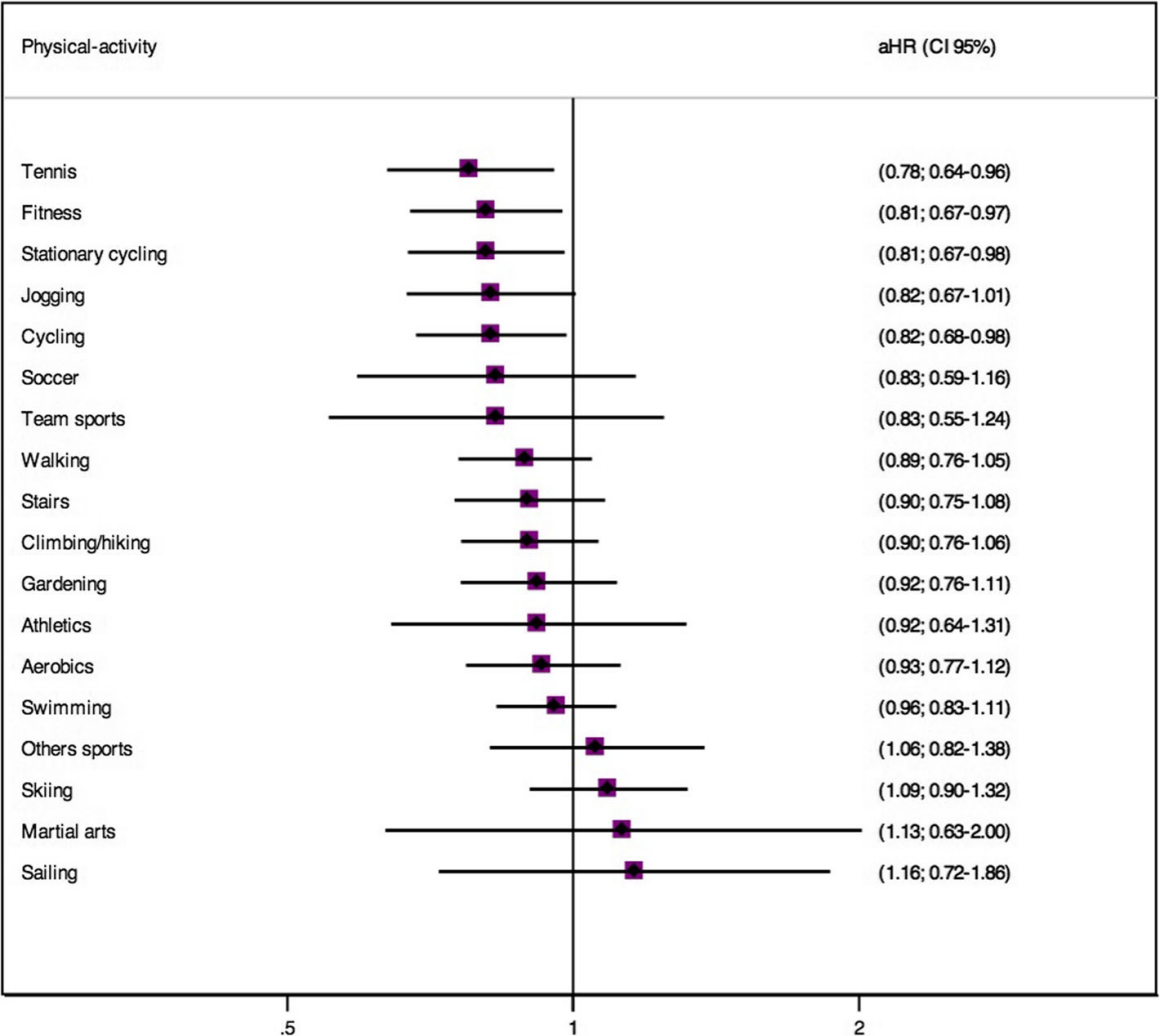
Multivariable-adjusted Hazard Ratio (aHR) across total LTPA by each type of PA and risk of depression. The SUN prospective cohort. Adjusted for sex, baseline body mass index, time sleeping, time nap, time TV, total energy intake, adherence to the Mediterranean Diet, alcohol intake, smoking pack years, educational level, hypertension, diabetes mellitus, cancer and changes in physical activity in the 2th and 4th year follow-up, with age and year of entering the cohort as stratification variables

Figure 3 shows the multivariable-adjusted Hazard Ratio (aHR) for depression across total LTPA by type of PA. Across the 17 different types of LTPA only tennis, fitness and cycling practices (normal cycling and stationary cycling) were significantly associated with lower depression risk.

**Fig. 4:**
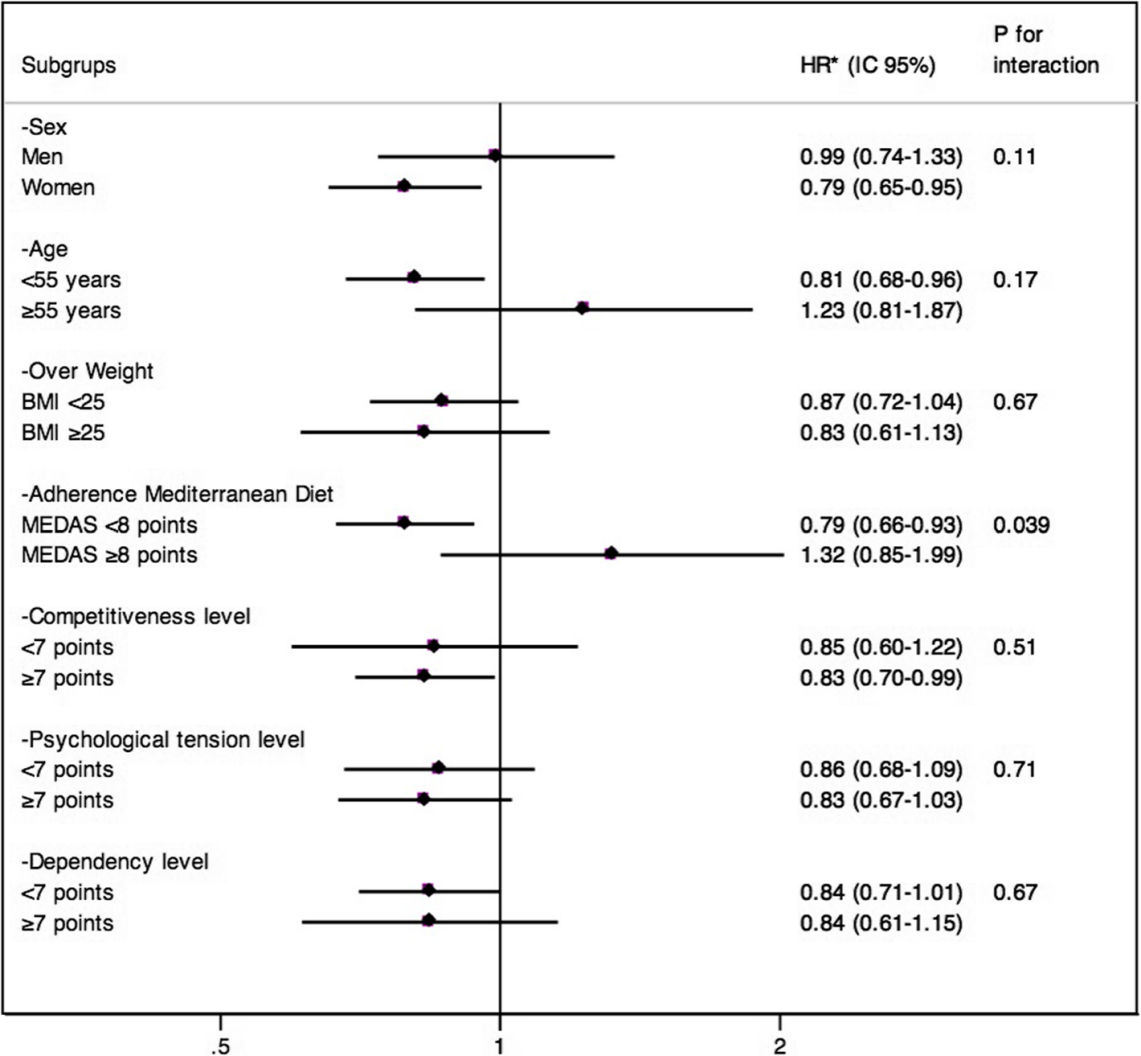
Stratified analysis between total Energy expenditure in LTPA and risk of depression. The SUN prospective cohort. Adjusted for sex, baseline body mass index, time sleeping, time nap, time TV, total energy intake, adherence to the Mediterranean Diet, alcohol intake, smoking pack years, educational level, hypertension, diabetes mellitus, cancer and changes in physical activity in the 2th and 4th year follow-up, with age and year of entering the cohort as stratification variables

In the stratified analyses we found an inverse association between total LTPA and depression in women, in participants younger than 55 years, in those with less of 8 points in the adherence of Mediterranean Diet questionnaire and in those with highest levels of competitiveness (Fig. 4). However, we only found a statistically significant multiplicative interaction between Mediterranean Diet and total LTPA (p interaction = 0.039).

To confirm a correct time sequence and ensure that LTPA temporally preceded depression incidence, supplemental analysis were performed where participants with a diagnosis of depression not only at baseline(Q0) but also in the first 2 years of follow (Q2) were not included and results remained similar. Participants who perform higher physical activity (LTPA> 20MET-h / week) had a depression HR of 0.80 (95% CI: 0.66–0.97) when compared to participants who performed less physical activity (< 10 MET-h / wk.)

## Discussion

In this longitudinal study, we found that higher levels of total LTPA (MET-h/wk), were associated with a lower risk of depression, consistently with previous studies, that report that PA is related with better mental health outcomes [13].. Our results show a modest reduction in the risk of depression (16%), but with a protective factor that is easy, simple and accessible to implement in the general population.

We also found that, while higher time spent in LTPA (hours/wk) was associated with lower incidence of depression, higher intensity (METS) was not related to depression risk. This is consistent with the notion that the type and duration of exercise are important, and that higher levels of PA beyond a certain high threshold do not confer additional protection [26].

In our cohort the inverse association between LTPA and depression was more marked in women, as previously reported [27]. Mediterranean lifestyle not only includes a pattern of food consumption but also other characteristics, such as socialization which has a demonstrated role in depression risk reduction [28]. In this context, when we analyzed the association between PA and depression stratifying by Mediterranean Diet we observed an interaction, the association of LTPA with depression was stronger in participants with lower adherences to Mediterranean diet. Perhaps LTPA adds little to the repeatedly reported inverse association of Mediterranean diet with depression [28, 29]. Once a level of lifestyle benefit is acquired, the additional beneficial effect of LTPA beyond diet might be negligible.

The relationship between LTPA and depression can be explained by several mechanisms, such as the anti-inflammatory and neurochemical known effect of PA [30]. The depressive process has an increase of pro-inflammatory cytokines, [31] with the consequent endothelial dysfunction, [32] and oxidative stress [33]. Also, the psychological factors that exercise has on self-body image, self-efficacy, or in the decrease of social isolation, can be mediators in the ethological pathway between PLTA and mental health [34]. Depressive disorder may lead to a reduction in physical activity (i.e., lower PA is a consequence and not a cause of depression), but the Mendelian randomization analysis suggest that lower PA does not seem to be a consequence of depression, but it is more likely to be a depression determinant [35].

We analyzed different types of sports in different environments, (indoor- outdoor, individual-collective), as different exposures or psychosocial mechanisms could be implicated. It has been suggested that improvements in mental health after LTPA could be related to social relations and social support, coming from LTPA done in teams, [36] but our results support that even sports such as cycling, fitness or jogging, that not necessary imply team collaboration also are associated with lower risk of depression. Even though the confidence intervals are wider for team sports, the results suggest that the effects seen in classical team sports such as football or basketball are actually lower than the effects found in individual sports. Our analyses do not seek to compare the effects of different types of sports on depression, but they do suggest that LTPA could have mental health benefits regardless the presence of socialization.

Although the relationship between depression and physical activity among adults has been studied as a preventive factor and as a treatment for mental illness, the current guidelines on PA proposed by WHO [17] and the scientific report of the Advisory Committee on Physical Activity Guidelines of 2018, [37] are nonspecific. The recommendations to achieve greater health benefits are given either by increasing the time or the energy. And as some studies show [10, 11] both cardio-metabolic health and reduction in mortality will benefit most from vigorous activities, while mental health is more influenced by the time spent in PA. In addition, in our study, we found that to reduce the risk of depression, it is necessary to increase the levels of moderate physical activity above 300 min per week or perform 150 min of vigorous physical activity per week, or an equivalent combination.

One possible methodological weakness of the SUN cohort is that both exposure and outcome were self-reported which could possibly result in information bias and inverse causality bias; however, several validation studies have proved the high quality of the information and self-reported data from SUN participants, and supplemental analysis performed to evaluate the possible inverse causality bias showed similar results. Depression diagnosis is a slow process, therefor some participants during the first SUN follow-up years, might have depression symptoms but without a clinical diagnosis, and it is possible that they have received the recommendation to increase their LTPA in order to improve their mood. To ensure a correct time sequence, between LTFA and depression, and confirm that the LTPA preceded in time to the depression diagnosis supplementary analyses were performed eliminating participants with a diagnosis of depression not only at baseline but also in the first follow up questionnaire (two first years of follow-up), obtaining similar results. Another limitation could be a possible reverse causation bias, which could lead to find less LTPA in those who do not tolerate PA because of a poor cardiopulmonary condition. To avoid this, we eliminated participants with prevalent CVD. The lack of repeated measures of LTPA, may produce misclassification and as metabolic equivalents were estimated against typical intensity metrics, the real PA intensity is difficult to measure (e.g., jogging can vary from almost walking to almost running from person to person) also leading to a possible misclassification bias. But this type of errors would tend to mitigate the differences across groups, leading the results to the null value; therefore, they do not affect the differences found. Physical activity data were obtained in the baseline questionnaire; but we have run our analyses, taking into account changes in physical activity reported during follow-up and have adjusted our models for these changes. Even though our sample is not representative, the validly to assess an association, does not require representativeness. And finally, as most SUN participants are young adults, the lower number of diseases can limit the statistical power, but it does not affect our findings.

The SUN study has a large sample size, a prospective design (which allows to evaluate the temporal sequence), and several previously published validation studies. One of the principal strengths of our cohort is the high retention rate (91%) and the long follow-up.

Among the different types of LTPA those who had a greater effect on depression prevention where sports than can easily be recommended to the general population and can be done inside or outside (normal cycling, and stationary cycling). Fitness, cycling and jogging are easy, affordable and widespread sports, which could be recommended to a wide range of age population.

These findings have implications on future planning of preventive intervention strategies focused on reducing depression. Depression is associated with a decrease in physical, cognitive and social functioning, morbidity and mortality generating a high social burden [38]. Identifying factors to prevent depression will have important implications for public health [39]. Preventing strategies should include Physical Activity programs, with special emphasis in the time spent in PA.

## Conclusion

This study found an association between higher levels of LTPA and lower risk of depression incidence. Total time of PA seems to be a greater protective factor for depression than the energy expenditure. Further studies should be conducted to confirm these data.

## Supplementary information

**Additional file 1.** Sources of variability and quantity of physical activity per type of activity. The SUN Project.

## Data Availability

The datasets used and analyzed during the current study are available from the corresponding author on reasonable request.

## Abbreviations

AHR: Multivariable-adjusted Hazard Ratio
CI: Confidence intervals
CVD: Cardiovascular disease
HR: Hazard ratio
LTPA: Leisure-time physical activity
QO: Baseline questionnaire
SD: Standard deviation
SUN: Seguimiento Universidad de Navarra
WHO: World Health Organization
WK: Week
