# Supplementary material for: Dimensions of leisure-time physical activity and risk of depression in the “Seguimiento Universidad de Navarra” (SUN) prospective cohort
Source: BMC Psychiatry. 2020 Mar 4;20:98. doi: 10.1186/s12888-020-02502-6 (PMC7055072; doi:10.1186/s12888-020-02502-6)
Supplement: Supplementary file 1 — Additional file 1. Sources of variability and quantity of physical activity per type of activity. The SUN Project. [file 12888_2020_2502_MOESM1_ESM.docx]

Sources of variability and quantity of physical activity per type of activity. The SUN Project

| **LTPA groups** | **Cumulative R²** | **% LTPA** |
| --- | --- | --- |
| Walking | 0.286 | 48% |
| Stairs | 0.313 | 12.7% |
| Climbing/hiking | 0.448 | 6.5% |
| Swimming | 0.506 | 5.8% |
| Fitness | 0.588 | 5.3% |
| Aerobics | 0.605 | 5.0% |
| Jogging | 0.731 | 4.6% |
| Tennis | 0.767 | 3.8% |
| Cycling | 0.805 | 3.6% |
| Gardening | 0.823 | 3.4% |
| Stationary cycling | 0.833 | 3.1% |
| Skiing | 0.857 | 2.9% |
| Other team sports | 0.888 | 2.5% |
| Soccer | 0.913 | 1.8% |
| Athletics | 0.985 | 1.8% |
| Team sports | 0.993 | 0.7% |
| Martial arts | 0.999 | 0.5% |
| Sailing | 1 | 0.2% |

LTPA: Leisure time physical activity
